# Evaluation of myocardial fibrosis by post gadolinium T1 measurement in patients with pulmonary hypertension

**DOI:** 10.1186/1532-429X-17-S1-P380

**Published:** 2015-02-03

**Authors:** Kimberly Kallianos, Gabriel Brooks, Charles Higgins, Karen Ordovas

**Affiliations:** 1 Department of Radiology and Biomedical Imaging, University of California, San Francisco, San Francisco, CA, USA; 2 Department of Cardiology, University of California, San Francisco, San Francisco, CA, USA

## Background

Cardiac magnetic resonance (MR) imaging can identify the presence of diffuse fibrosis in patients with myocardial diseases using T1 mapping. We evaluated the post-gadolinium T1 relaxation time in milliseconds (ms) in patients with pulmonary hypertension compared to controls to investigate for the presence of diffuse myocardial fibrosis in this patient population.

## Methods

Patients (n=19) with a clinical diagnosis or imaging findings of pulmonary hypertension who underwent cardiac MR between 1/1/2009 and 9/30/2014 were identified. Controls (n=10) were referred for cardiac MR evaluation to rule out ARVD due to family history or palpitations, but with a normal cardiac MR examination. Both patients and controls underwent cardiac MR with delayed gadolinium enhancement. Post-contrast lock-locker inversion recovery sequences were obtained approximately 15 minutes following administration of gadolinium. T1 values were measured in the ventricular septum by placing a region of interest (ROI) that was confined to the myocardium using a semiautomated method. Manual correction was used to adjust ROIs that included the blood-pool. Statistical analysis was performed using Kruskal-Wallis equality-of-populations rank test to compare the patient and control groups due to data skewness.

## Results

There was no significant difference in the age of patients versus controls, with a mean patient age of 56 ± 11 years, and a mean control age of 48 ± 18 years (p=0.13). Males comprised 53% of the patient group and 30% of the control group. Of the nineteen patients with pulmonary hypertension, two had primary pulmonary hypertension, while the remaining patients had secondary pulmonary hypertension. The main causes of secondary pulmonary hypertension in this group were heart failure, sarcoidosis, scleroderma, lupus, and congenital heart disease. The median ventricular septal myocardial T1 value in controls was 388 ms (interquartile range 84 ms) while the median T1 value in patients was 343 ms (interquartile range 71 ms). The ventricular septal myocardial T1 values were significantly lower in patients with pulmonary hypertension compared to controls (p=0.026), suggesting the presence of diffuse myocardial fibrosis.

## Conclusions

The presence of significantly lower ventricular septal T1 values in patients with pulmonary hypertension as a marker of fibrosis illustrates the ability of cardiac MR to assess for diffuse myocardial disease in this population. Cardiac MR may therefore serve as a non-invasive tool to identify early left ventricular abnormalities in these patients with the potential to inform prognosis and evaluate effectiveness of treatment strategies in patients with pulmonary hypertension.

## Funding

None.

**Figure 1:**
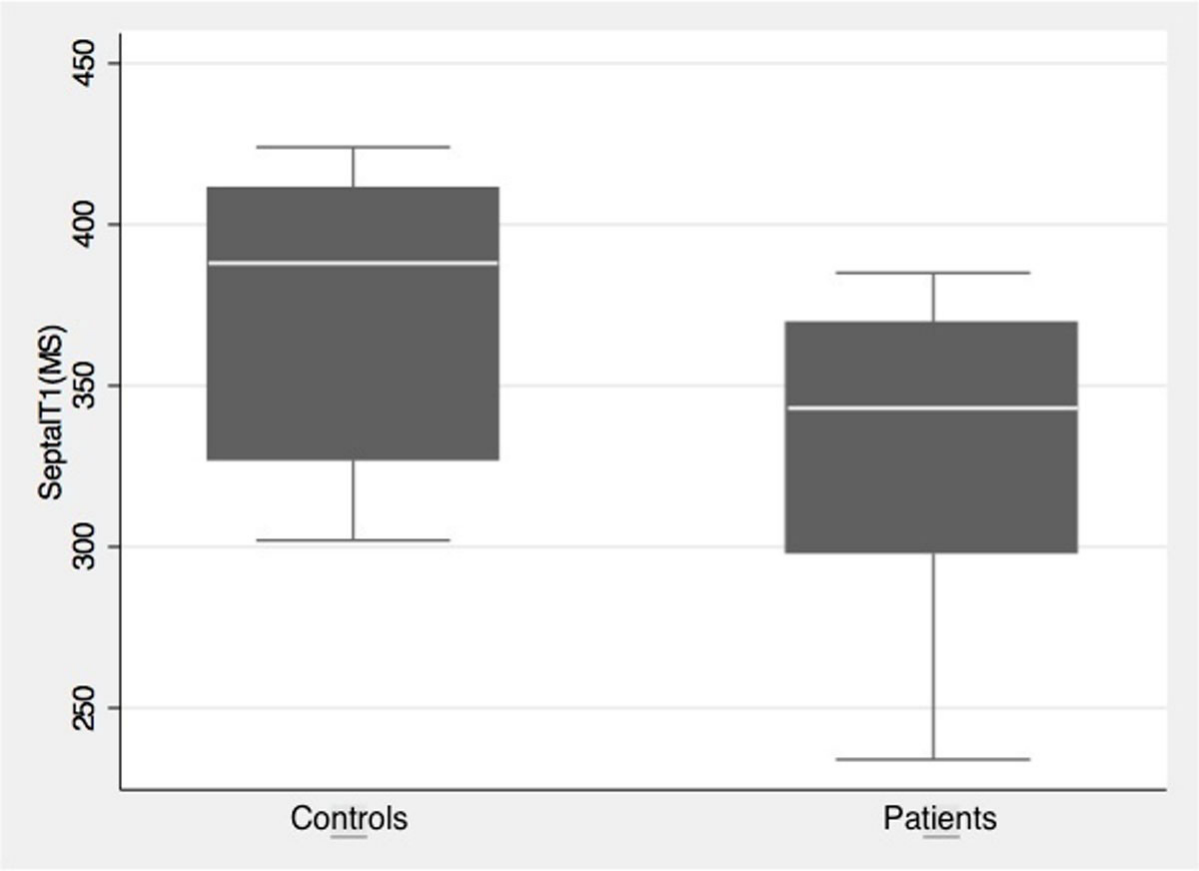
Post-gadolinium ventricular septal myocardial T1 values in controls versus patients with pulmonary hypertension.

